# Neglected immunoregulation: M2 polarization of macrophages triggered by low‐dose irradiation plays an important role in bone regeneration

**DOI:** 10.1111/jcmm.17721

**Published:** 2023-03-16

**Authors:** Shaoqing Chen, Su Ni, Chun Liu, Mu He, Yiwen Pan, Pengfei Cui, Cheng Wang, Xinye Ni

**Affiliations:** ^1^ The Affiliated Changzhou No. 2 People's Hospital of Nanjing Medical University Changzhou China; ^2^ Jiangsu Province Engineering Research Center of Medical Physics Changzhou China; ^3^ School of Pharmacy Changzhou University Changzhou China

**Keywords:** bone regeneration, irradiation, macrophage polarity, osteoblast proliferation

## Abstract

Current studies have found that low‐dose irradiation (IR) can promote bone regeneration. However, mechanism studies of IR‐triggered bone regeneration mainly focus on the effects of osteoblasts, neglecting the role of the surrounding immune microenvironment. Here in this study, in vitro proliferation experiments showed that low‐dose IR ≤2 Gy could promote the proliferation of bone marrow mesenchymal stem cells (BMSCs), and qRT‐PCR assay showed that low‐dose IR ≤2 Gy could exert the M2 polarization of Raw264.7 cells, while IR >2 Gy inhibited BMSC proliferation and triggered M1 polarization in Raw264.7 cells. The ALP and mineralized nodules staining showed that low‐dose IR ≤2 Gy not only promoted osteoblast mineralization through IR‐triggered osteoblast proliferation but also through M2 polarization of Raw264.7 cells, while high‐dose IR >2 Gy had the opposite effect. The co‐incubation of BMSC with low‐dose IR irradiated Raw264.7 cell supernatants increased the mRNA expression of BMP‐2 and Osx. The rat cranial defects model revealed that low‐dose IR ≤2 Gy gradually promoted bone regeneration, while high‐dose IR >2 Gy inhibited bone regeneration. Detection of macrophage polarity in peripheral blood samples showed that low‐dose IR ≤2 Gy increased the expression of CD206 and CD163, but decreased the expression of CD86 and CD80 in macrophages, which indicated M2 polarization of macrophages in vivo, while high‐dose IR had the opposite effect. Our finding innovatively revealed that low‐dose IR ≤2 Gy promotes bone regeneration not only by directly promoting the proliferation of osteoblasts but also by triggering M2 polarization of macrophages, which provided a new perspective for immune mechanism study in the treatment of bone defects with low‐dose IR.

## 1 INTRODUCTION

Bone defects are still a medical concern in recent decades. Millions of bone fixation are performed each year. Bone autografts and allografts, or biologically inert metallic devices‐based bone fixation, are the gold standard for the treatment for large bone defects.^1^, ^2^ With the development of biomaterials, many synthetic and natural biomaterials with good biocompatibility and biodegradability are explored for the application of implanted scaffolds, such as collagen,^3^ calcium phosphate,^4^ hydroxyapatite^5^, ^6^ and silica.^7^ There is no denying that these implants also bring some inevitable side effects when they work. Subsequent surgical removal is often needed for metal bone‐fixation devices.^8^ The use of bone autografts or allografts often carries a risk of disease transmission from the donor material, additional donor site morbidity and limited availability compared to the need.^9^ The biodegradable implanted scaffolds may face biological toxicity, bacterial infection and inflammatory responses.^10^

In recent years, more and more researchers have found that in addition to implantation, irradiation (IR) has been found to play a role in bone regeneration. Deloch et al.^11^ showed a positive impact of 0.1 and 0.5 Gy on bone formation of healthy osteoblasts. Wright et al.^12^ reported that 2 Gy of IR could result in local and systemic bone loss in C57BL/6 mice. Chen et al.^13^ showed that 0.5 Gy of IR promoted the fracture repair in Sprague Dawley (SD) rats. In the work of Hu et al.,^14^ x‐ray at 2 Gy decreased the mineralization effect of OCT‐1 cells after a single IR. Sun et al.^15^ showed that a 2 Gy local X‐IR in rats led to an inhibition of osteogenic differentiation. In contrast, the same cell line subjected to the same dose exerted only time‐dependent cell cycle arrest without significant effects on the proliferation and differentiation effect,^16^ while another study further suggested that 2 Gy of x‐ray not only increased differentiation and mineralization potential of calvarial osteoblasts but also upregulated the expression of many related cytokines, including alkaline phosphatase (ALP), osteopontin (OPN) and osteocalcin (OCN) during the process.^17^ Liu et al.^18^ reported that 6 Gy of IR promoted ROS generation by mediating upregulation of miR‐22, which in turn promoted apoptosis of bone marrow mesenchymal stem cells (BMSCs).

These studies mainly illustrate the effect of IR on osteoblasts (OBs) and osteoclasts (OCs) in bone tissue. In addition to OBs and OCs, the bone microenvironment also includes various types of cells that affect bone remodelling.^19^ It was reported that IR affects not only OBs and OCs but also other cells in the bone microenvironment.^20^ IR affects bone vasculature, while bone vasculature has a vital effect on bone homeostasis as a transporter of nutrients and oxygen.^21^ Studies showed reduced blood flow in irradiated bones in mice and rats after varying doses of IR.^22^ IR also affects immune microenvironment in the bone^23^; however, the detailed osteoimmunological mechanism has not been revealed. The effect of IR on bone regeneration is not only dependent on changes in OBs and OCs biology, but also closely related to changes in the overall bone microenvironment. Since bone destruction or remodelling is a dynamic process, and some studies have also explored the effect of low‐dose IR on bone and bone formation‐related cells, there are more related studies that are worth exploring.

Macrophages, as a critical component of the innate immune system, have been demonstrated to exert important regulatory roles in bone homeostasis and repair.^24^, ^25^ M1‐polarized macrophages play an important role in the initiation and development of inflammation, and produce effector molecules such as reactive oxygen species (ROS), inducible nitric oxide synthase (iNOS), and inflammatory cytokines such as IL‐1β, IL‐6 and TNF‐α. Due to their inflammatory properties, chronic activation of M1 macrophages leads to tissue damage. On the other hand, M2‐polarized macrophages secrete anti‐inflammatory cytokines such as IL‐4 and IL‐10. In vivo, these major isoforms are not strictly formed, but are interchangeable.^26^, ^27^ It was reported that bone repair is positively associated with M2 macrophage function,^28^ and regulated M2 macrophages showed a preventive effect on bone loss in murine periodontitis models.^29^ Recently, regulated M2 macrophage polarization using scaffold implantation or exosome was found to significantly enhance bone formation or inhibit periodontal bone loss in animal models.^30^, ^31^ In recent decades, studies have come to a consensus that macrophages subjected to ≤1 Gy of IR treatment were likely to be prone to M2 polarization (anti‐inflammatory), while >2 Gy of IR was more prone to enhance M1 polarization (pro‐inflammatory) of macrophages.^32^, ^33^ Therefore, the impact of low‐dose IR on macrophages and the following impact on bone repair, as a long‐neglected mode of action, should be considered.

Here in this study, we explored the effect of low‐dose IR ≤2 Gy on OB proliferation and macrophage polarity, revealing the effect of macrophage polarization on bone regeneration. Rats with cranial defects were used as the animal model to study the bone regeneration effect of low‐dose IR on bone defects. Our study is helpful to understand the detailed osteoimmunological mechanism of bone regeneration induced by low‐dose IR and provides guidance for the development of bone defect‐related diseases and osteoimmunology.

## 2 MATERIALS AND METHODS

### 2.1 Materials

Lipopolysaccharides (LPS) and BeyoClick™ EdU Cell Proliferation Kit with Alexa Fluor 488 was supplied by the Beyotime. NBT/BCIP tablets were supplied by Roche Diagnostics GmbH. Alizarin Red, Fluorescein diacetate (FDA) and Propidium Iodide (PI) were obtained from Solarbio Life Sciences. RNA‐Quick Purification Kit was obtained from ES Science. HiScript® II Q RT SuperMix for qPCR and AceQ® qPCR SYBR Green Master Mix were provided by Vazyme. CD68 monoclonal antibody (ED1), CD80 (B7‐1) monoclonal antibody (3H5) and Goat anti‐rabbit IgG (H+L) secondary antibody, PE‐Cyanine 5.5 were supplied by Invitrogen. CD206 antibody was obtained from Santa Cruz Biotechnology, Recombinant Anti‐CD163 antibody was supplied by Abcam. RUNX2 Rabbit mAb was supplied by Cell Signaling Technology Inc. PE‐anti rat CD86 antibody, APC goat anti‐mouse IgG antibody and APC/Cyanine 7 streptavidin were obtained from Biolegend.

### 2.2 Methods

#### 2.2.1 Cell culture

Raw264.7 cells were cultured in Dulbecco's modified Eagle's medium (DMEM, high glucose) supplemented with 10% foetal bovine serum in a 37°C incubator. The trypsin–EDTA solution was used to trypsinize the cells. Rat BMSCs were cultured with DMEM (low glucose).

#### 2.2.2 Cell proliferation and viability

The MTT assay was employed to determine the cell proliferation of BMSCs under different doses of IR (0, 0.5, 1, 2, 4 Gy). In brief, BMSCs were seeded into 96‐well plates (6 × 10^3^ cells/well) and incubated for 12 h, then treated with different doses of IR (0, 0.5, 1, 2, 4 Gy) by x rays using the linear accelerator Infinity (Elekta Corporation). The dose rate was 600 MU/min and the energy was 6 MV. After IR the cells were returned to the incubator for further cultivation (24, 48 and 72 h). MTT solution (5 mg/mL, 20 μL) was added to each well at determined time intervals for another 4 h of incubation. The medium was removed, and 200 μL of DMSO was added, which was used to dissolve the formazan crystals. The whole plate was shaken at 37°C for 30 min, then subjected to measurement at 570 nm using a spectrophotometer (Epoch, BioTek). Untreated cells were selected as blank control.

Bone marrow mesenchymal stem cells were seeded into 24‐well plates (3 × 10^4^ cells/well) and allowed to grow for 12 h. Then cells were treated with different doses of IR (0, 0.5, 1, 2, 4 Gy) and returned to the incubator for further cultivation of 24, 48, 72 h, respectively. FDA (50 μg/mL) and PI (5 μg/mL) were added to the medium for the staining of live and dead cells. Afterwards, cells were washed gently by PBS twice and subjected to observation using an inverted fluorescence microscope (iX71, Olympus).

Bone marrow mesenchymal stem cells were seeded into 6‐well plates (5 × 10^4^ cells/well) and allowed to grow for 12 h. Then cells were treated with different doses of IR (0, 0.5, 1, 2, 4 Gy) and returned to the incubator for further cultivation of 72 h. After incubation, BeyoClick™ EdU Cell Proliferation Kit with Alexa Fluor 488 was applied according to the manufacturer's instructions. The samples were detected by the Flow cytometer (BD FACSCanto II).

#### 2.2.3 Polarity detection of Raw264.7 cells

Raw264.7 cells were seeded in 6‐well plates (2 × 10^5^ per well), and incubated for 24 h. After culturing, the cells were stimulated by lipopolysaccharide (LPS, 1 μg/mL) for 24 h, rinsed with PBS, replaced with fresh culture medium, then irradiated with various doses (0, 0.5, 1, 2, 4 Gy) of IR and continued to incubate for 12 h. Messenger RNA (mRNA) was extracted using an RNA‐quick purification kit. Then, the RNA samples were added with SuperMix, reverse‐transcribed into complementary DNA at 50°C for 15 min, 85°C for 5 s. The qRT‐PCR assay was conducted within the mixture using AceQ® qPCR SYBR Green Master Mix. Finally, the mRNA expression of macrophage M1 polarization marker genes IL‐1β, iNOS, and M2 polarization marker genes BMP2, CD206, were quantified using a real‐time PCR detection system (ViiA7, ThermoFisher Scientific). The relative mRNA expression levels of the target genes were normalized using GAPDH, and gene expression was calculated using the 2^−ΔΔCT^ method. Primers used in this study are displayed in the Table S1.

#### 2.2.4 In vitro osteogenic differentiation tests

To differentiate BMSCs into osteoblasts, different extracts were used to make up an osteoblastic induction medium (OIM) supplemented with 2 mM β‐glycerophosphate, 10 nM dexamethasone and 100 μM ascorbic acid. Standard growth medium was regarded as the control. To study the effect of macrophage polarization on the osteogenesis ability of BMSCs, the supernatants of Raw264.7 cells after irradiation with different doses after cultured for 12 h were collected and co‐cultured with BMSCs.

Six‐well plates were added with 0.1% gelatin solution and placed in the incubator for 1 h, then the gelatin solution was removed. Afterwards, BMSCs were seeded into 6‐well plates (1 × 10^4^ cells/well, marked as Day 1). On Day 3, the primary medium was replaced by a different medium, and the arranged groups were listed as follows: (1) Group I: low glucose DMEM; (2) Group II: OIM; (3) Group III‐VI: OIM + 0.5, 1, 2, 4 Gy IR, respectively; (4) Group VII: OIM + supernatant of Raw264.7 cells (without IR); (5) Group VIII‐XI: OIM + 0.5, 1, 2, 4 Gy IR + supernatant of Raw264.7 cells (with the corresponding dose of IR), respectively. The medium was replaced every 3 days for 2 weeks. On Day 13, the cells were fixed with 4% paraformaldehyde for 15 min. NBT/BCIP colour substrate solution was prepared by adding one tablet into 10 mL water. The cells were stained with NBT/BCIP colour substrate solution for 20 min at 37°C, and observed under an optical microscopy. At Day 21, the cells were fixed with 4% paraformaldehyde for 15 min, and stained with Alizarin Red for 20 min at 37°C. Then the samples were washed with PBS to remove nonspecific staining and directly observed by the optical microscopy.

#### 2.2.5 Expression of osteogenic genes

Raw264.7 cells were seeded in 6‐well plates (5 × 10^4^ per‐well), and incubated for 48 h. After culturing, the cells were stimulated by LPS (1 μg/mL) for 24 h, rinsed with PBS, replaced with fresh culture medium, then irradiated with various doses (0, 0.5, 1, 2, 4 Gy) of IR, and continued to incubate for 12 h, and the supernatants were collected for later use (called supernatants 1). After suction of supernatant 1, the cells in the plates were added with LPS (1 μg/mL) for 24 h, rinsed with PBS, replaced with fresh culture medium for additional culture of 12 h, and the supernatants were also collected for later use (called supernatants 2).

Six‐well plates were added with 0.1% gelatin solution and placed in the incubator for 1 h, then the gelatin solution was removed. Afterwards, BMSCs were seeded into 6‐well plates (1 × 10^4^ cells/well) and cultured in OIM for 24 h, then added with supernatants 1 or supernatants 2 for 7 days. After that, messenger RNA (mRNA) was extracted using an RNA‐quick purification kit. Then, the RNA samples were added with SuperMix, reverse‐transcribed into complementary DNA at 50°C for 15 min, 85°C for 5 s. The qRT‐PCR assay was conducted within the mixture using AceQ® qPCR SYBR Green Master Mix. Finally, the mRNA expression of BMP‐2 and Osterix (Osx) were quantified using a real‐time PCR detection system as above. The relative mRNA expression levels of the target genes were normalized using GAPDH, and gene expression was calculated using the 2^−ΔΔCT^ method. Primers used in this study are displayed in the Table S1.

#### 2.2.6 In vivo osteogenesis evaluation

All animal experiments were approved by the Nanjing Medical University Ethics Committee. Male Sprague Dawley (SD) rats (200–250 g) were obtained from Suzhou Sinocell Technology Ltd.

The rats were conducted to drilling operations to construct cranial defect models. Briefly, the rats were first anaesthetized by intraperitoneal injection of 1% pentobarbital sodium. Subsequently, an incision with the appropriate size was made over the scalp with a scalpel for the exposure of cranium. The periosteum was splitted for the exposure of underlying bone. A trephine drill was used to create a 5‐mm‐diameter cranial defect on both sides of the midline under continuous sterile saline irrigation. Finally, the incisions were sutured after drilling.

The rats were randomly divided into five groups after the operation (*n* = 3). The following treatments were conducted the next day: Group I did not receive any therapy, Group II‐V received 0.5, 1, 2 and 4 Gy total body IR by the linear accelerator Infinity (Elekta Corporation), respectively. After 4 weeks of feeding, the rats were sacrificed, the craniums of rats were removed and fixed in formalin. Micro‐computed tomography (Micro‐CT, SkyScan 1176) was used to evaluate the craniums with the following settings: voltage: 65 kV, electricity: 385 μA, Al filter: 0.5 mm. The two‐dimensional (2D) and three‐dimensional (3D) structures of the cranium were reconstructed using Mimics software. Bone volume/tissue volume (BV/TV) and trabecular thickness (Tb.Th) were analysed by CT‐Analyser software.

#### 2.2.7 Immunohistochemical staining

Immunohistochemical staining was performed to evaluate the expression of osteogenesis specific biomarkers and polarization‐related markers. The immunohistochemistry of runt‐related transcription factor 2 (RUNX2) was performed as follows: the fixed cranium samples were decalcified in 5% EDTA‐Na2 (pH = 7.3), followed by dehydrating, embedding in paraffin and making paraffin sections. Then the sections were permeated with Triton X‐100, and cultured with 5% goat serum for 1 h. After that, the sections were incubated with the primary antibody of RUNX2 (1:100 dilution) for 12 h at 4°C. Finally, the secondary antibody was added and incubated for 2 h. Similarly, the immunohistochemical staining of CD86 and CD163 was a similar procedure. Finally, the samples were observed by a light microscopy.

#### 2.2.8 Macrophage polarity in the blood

According to the above methodology, SD rats were modelled with cranial defect, and divided into the following five groups (*n* = 5) for IR on the second day: Group I served as blank controls without irradiation treatment, Group II–V received 0.5, 1, 2 and 4 Gy IR, respectively. The day of IR was recorded as Day 0, and blood samples were taken from the orbit of each rat on Day 1, 8 and 14. Peripheral blood mononuclear cells (PBMC) were separated from the blood samples by Lymphoprep according to the protocols. Separated samples were first incubated with 10% mouse serum for Fc block (30 min), labelled with CD206 antibody and recombinant anti‐CD163 antibody for 30 min, then APC Goat anti‐mouse IgG antibody and PE‐Cyanine 5.5 were applied. After staining of CD163 and CD206, the samples were treated with CD80 monoclonal antibody and PE‐anti rat CD86 antibody for 30 min, then stained with APC/Cyanine 7 streptavidin. After the labelling of cell surface antibody, the samples were fixed with 4% paraformaldehyde, permeated with intracellular staining perm wash buffer (1X) and stained with PE‐anti rat CD86 antibody. Flow cytometry (BD FACSCanto II) was conducted to determine CD80, CD86 positive (CD80^+^CD86^+^), and CD163, CD206 positive (CD163^+^CD206^+^) peripheral blood monocytes in the blood samples. The sampling and detection process of the polarity changes of peripheral blood monocytes in healthy rats after IR were provided by supporting information.

#### 2.2.9 Statistical analysis

All data are presented as the mean value ± standard deviation from at least three independent measurements. Comparisons among multiple groups were analysed using the one‐way anova with Tukey's multiple comparisons test. The statistical comparisons were performed using GraphPad Prism 8 (version 7; GraphPad Software, Inc.). Mean differences with *p* < 0.05 were considered statistically significant. The corresponding markers in the figures are defined as *p < 0.05, **p < 0.01 and ***p < 0.001, respectively.

## 3 RESULTS AND DISCUSSION

### 3.1 Low‐dose IR promotes the proliferation of BMSCs

**FIGURE 1:**
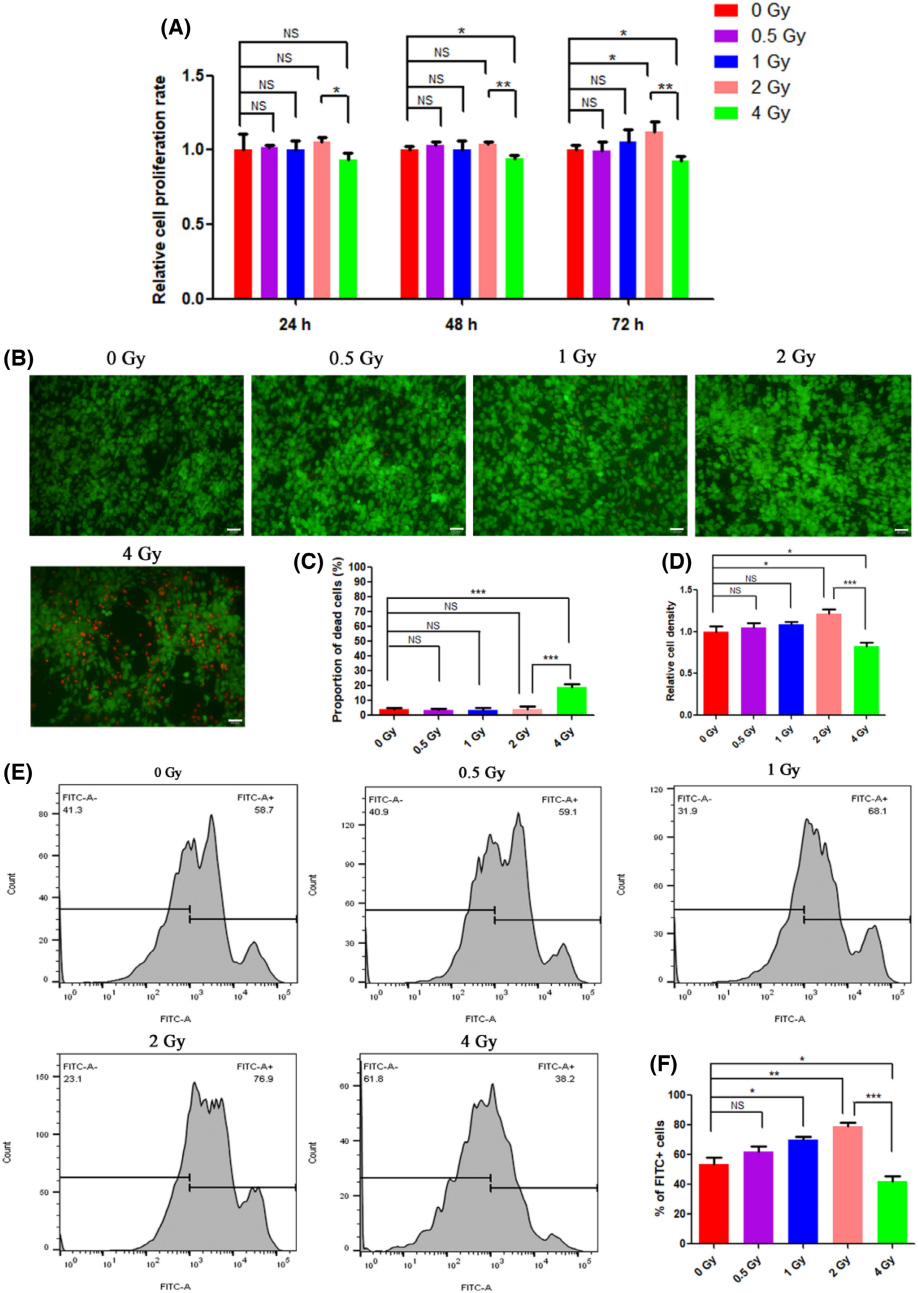
The effects of different doses of IR on the proliferation of BMSCs. (A) MTT assay of BMSCs at 24, 48 and 72 h post‐IR. (B) BMSCs were stained with FDA/PI at 72 h post‐IR. Green: FDA, red: PI. (C) Semi‐quantitative result of proportion of dead cells. (D) Semi‐quantitative result of relative cell density. (E) Flow cytometry results of the EdU assay. (F) Semi‐quantitative result of proportion of FITC^+^ cells. NS: no significant difference, *p < 0.05, **p < 0.01. The error bars in the graphs represent the standard deviation (*n* = 3). Scale bar, 50 μm.

Previous reports have all agreed that IR can influence the proliferation of osteoblasts, but the reported dose and final effects (positive or negative) remained controversial.^34^, ^35^, ^36^ To study the impact of IR doses on the proliferation of BMSCs, the cell viability study and live/dead staining were conducted. After being treated with different doses (0–4 Gy) of IR, BMSCs were allowed to grow for another 24, 48 or 72 h, and the cell viability were monitored using the MTT assay. As shown in Figure 1A, at 24 h post‐IR, there was almost no difference in cell viability among low‐dose IR ≤2 Gy groups, when the IR dose continued to increase to 4 Gy, the relative cell proliferation rate decreased to 0.88‐fold of that of the 2 Gy group. At 48 h, the cell proliferation rate of the 4 Gy group was significantly inhibited, which reduced to 0.94‐fold of that of the 0 Gy group and 0.91‐fold of that of the 2 Gy group. It was noted that extended incubation time to 72 h showed more significant differences. In particular, BMSCs subjected to 2 Gy IR showed the highest BMSC proliferation profile and had a significant difference compared to the 0 Gy control group, the relative cell proliferation rate of which increased to 1.13‐fold of that of the 0 Gy group. While 4 Gy group exhibited more significant differences compared with other groups, which had an obvious decline in cells. The effect of IR on the proliferation of macrophages by the MTT assay was also conducted. As shown in Figure S1, there was no significant differences in the proliferation of macrophages between various doses of IR.

In the live/dead staining assay performed at 72 h post‐incubation, as shown in Figure 1B–D, the 4 Gy group exhibited 19.3% of dead cells in the view. The cell density of the 4 Gy group was 0.83 times that of the 0 Gy group. While other groups showed high cell viability with almost no dead cells observed, particularly the cell density in the 2 Gy group was the highest among all groups, which was 1.21 times that of the 0 Gy group.

The results of EdU Cell Proliferation Kit with Alexa Fluor 488 detection (Figure 1E,F) showed the similar trend. From 0 to 2 Gy, the proportion of FITC^+^ cells gradually increased from 53.6% in the 0 Gy group to 79.2% in the 2 Gy group with the increase of IR dose. When the IR dose was improved to 4 Gy, the proportion of FITC^+^ cells decreased to 42%. These results suggests that low‐dose IR ≤2 Gy promotes the proliferation of BMSCs, while high‐dose IR >2 Gy inhibits the proliferation of BMSCs.

### 3.2 Low‐dose of IR promotes M2 polarization of macrophages

**FIGURE 2:**
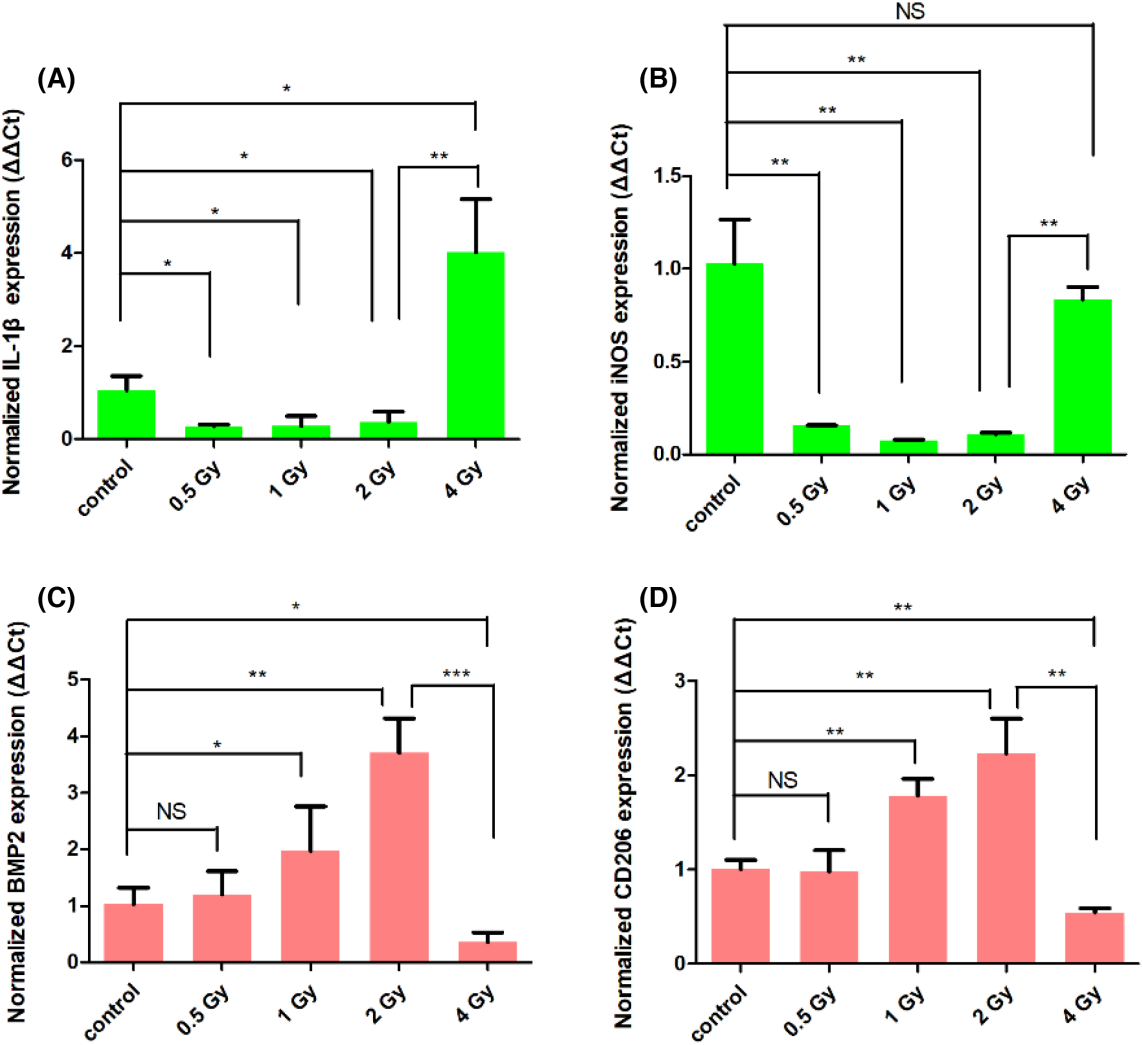
Raw264.7 cells were exposed to LPS inflammatory stimulation, then irradiated by different doses of IR, and cultured for 24 h. RT‐PCR was used to determine the intracellular mRNA expression of M1 markers (A) IL‐1β, (B) iNOS and M2 markers (C) BMP2, (D) CD206. NS: no significant difference, *p < 0.05, **p < 0.01, ***p < 0.001. The error bars in the graphs represent the standard deviation (*n* = 3).

As previous studies revealed that macrophages reside during all stages of fracture repair, which also contribute significantly to determine the destiny of repair process.^37^, ^38^ M2 polarization of macrophages could improve the repair outcome with reduced recuperation time.^39^, ^40^ Some studies have reported that macrophages subjected to ≤1 Gy of IR treatment were likely to become M2 polarization (anti‐inflammatory), while IR >2 Gy was more prone to enhance M1 polarization (pro‐inflammatory) of macrophages.^32^, ^33^ Therefore, there is a high potential that low‐dose IR can indirectly navigate the bone repair process by affecting macrophages polarization. To monitor the effect of IR dose on the polarization of macrophages in vitro, Raw264.7 cells were first treated with LPS to construct an in vitro inflammation model. Then the cells were subjected to different doses (0–4 Gy) of IR, followed by quantification of mRNA of M1/M2 markers using RT‐PCR. IL‐1β (Figure 2A) and iNOS (Figure 2B) were selected as M1 polarization markers, while BMP2 (Figure 2C) and CD206 (Figure 2D) were selected as M2 polarization markers. As shown in Figure 2, low‐dose IR ≤2 Gy reduced the expression of M1 polarization‐related mRNA and increased the expression of M2 polarization‐related mRNA as the IR dose increased. Compared with the 0 Gy control group, the 0.5 Gy group had the expression of IL‐1β and iNOS decreased to 0.27‐ and 0.16‐fold, with inconspicuous change in the expressions of BMP2 and CD206. While the 1 Gy group had the expression of IL‐1β and iNOS decreased to 0.28‐ and 0.07‐fold, and the expression of BMP2 and CD206 increased to 1.97‐ and 1.78‐fold. In the 2 Gy group, the expression of IL‐1β and iNOS were 0.37‐ and 0.11‐fold that of the control group and the expression of BMP2 and CD206 were 3.71‐ and 2.23‐fold that of the control group. While high‐dose IR >2 Gy exhibited an opposite result, for the 4 Gy group, the expression of IL‐1β and iNOS were 4.0‐ and 0.83‐fold that of the control group, and the expression of BMP2 and CD206 were 0.36‐ and 0.54‐fold that of the control group. These results indicate that low‐dose IR ≤2 Gy can promote the polarization of macrophages into M2 type, while IR >2 Gy can promote the polarization of macrophages into M1 type. Meanwhile we have checked the proportion of M1/M2 in Raw264.7 cells (Figure S2). The flow cytometry results showed when Raw264.7 was stimulated by LPS, the proportion of M1 macrophages was 84.3%, and the proportion of M2 macrophages was only 0.9%. When the IR dose increased, the proportion of M1 decreased and M2 increased gradually. At 2 Gy, the proportion of M2 was 12.6% and M1 was 34.7%. However, when the dose continued to increase to 4 Gy, the proportion of M2 macrophages significantly decreased (0.8%) but M1 macrophages increased (88.5%).

### 3.3 Low‐dose IR‐induced osteoblast proliferation and macrophage M2‐polarization promote osteogenesis

**FIGURE 3:**
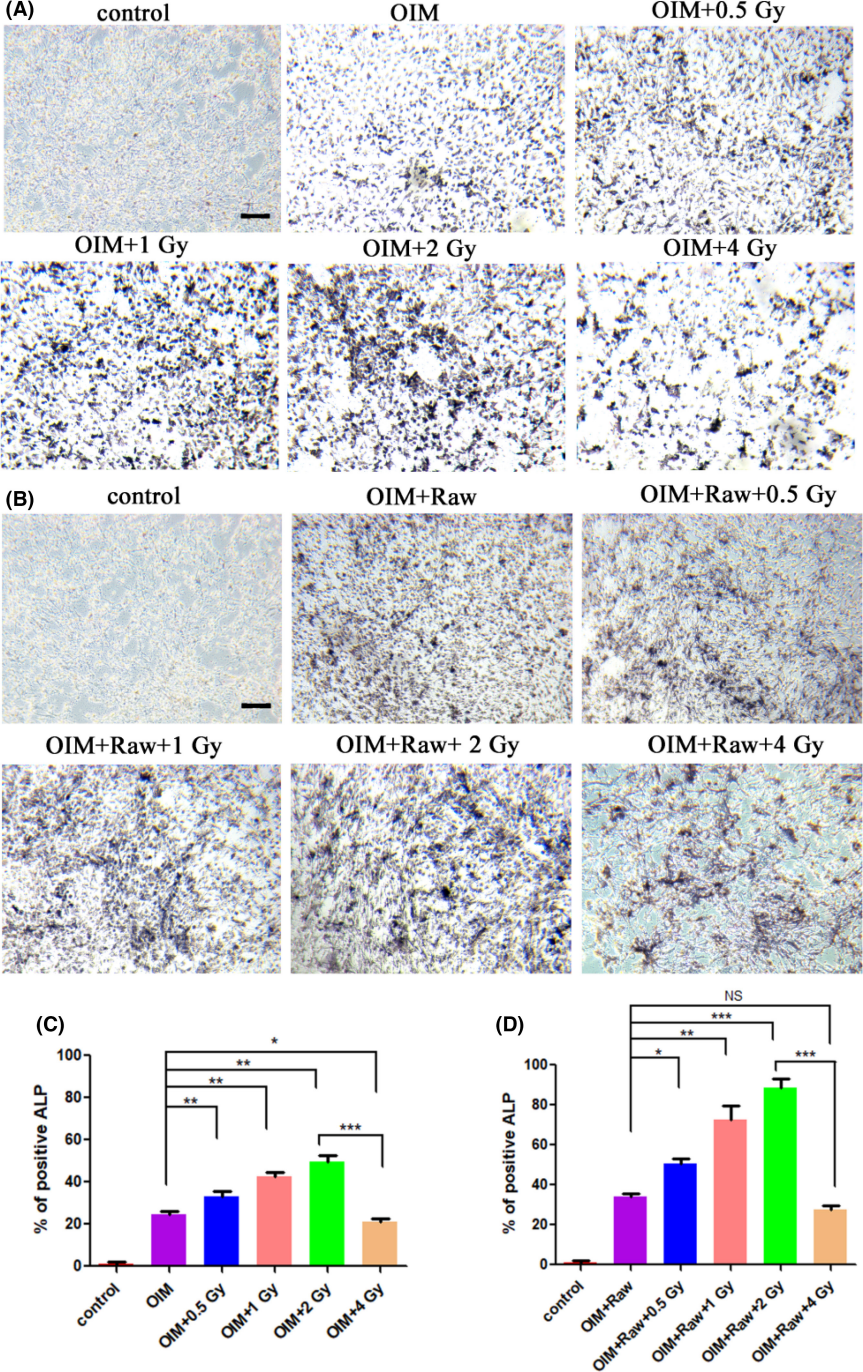
ALP staining of BMSCs with different treatments at Day 13. (A) BMSCs treated with OIM and different doses of IR. (B) BMSCs treated with OIM, different doses of IR, and supernatants of Raw264.7 cells irradiated with corresponding doses IR. (C) Semi‐quantitative of figures in (A). (D) Semi‐quantitative of figures in (B). Scale bar, 100 μm. NS: no significant difference, *p < 0.05, **p < 0.01, ***p < 0.001. The error bars in the graphs represent the standard deviation (*n* = 3).

**FIGURE 4:**
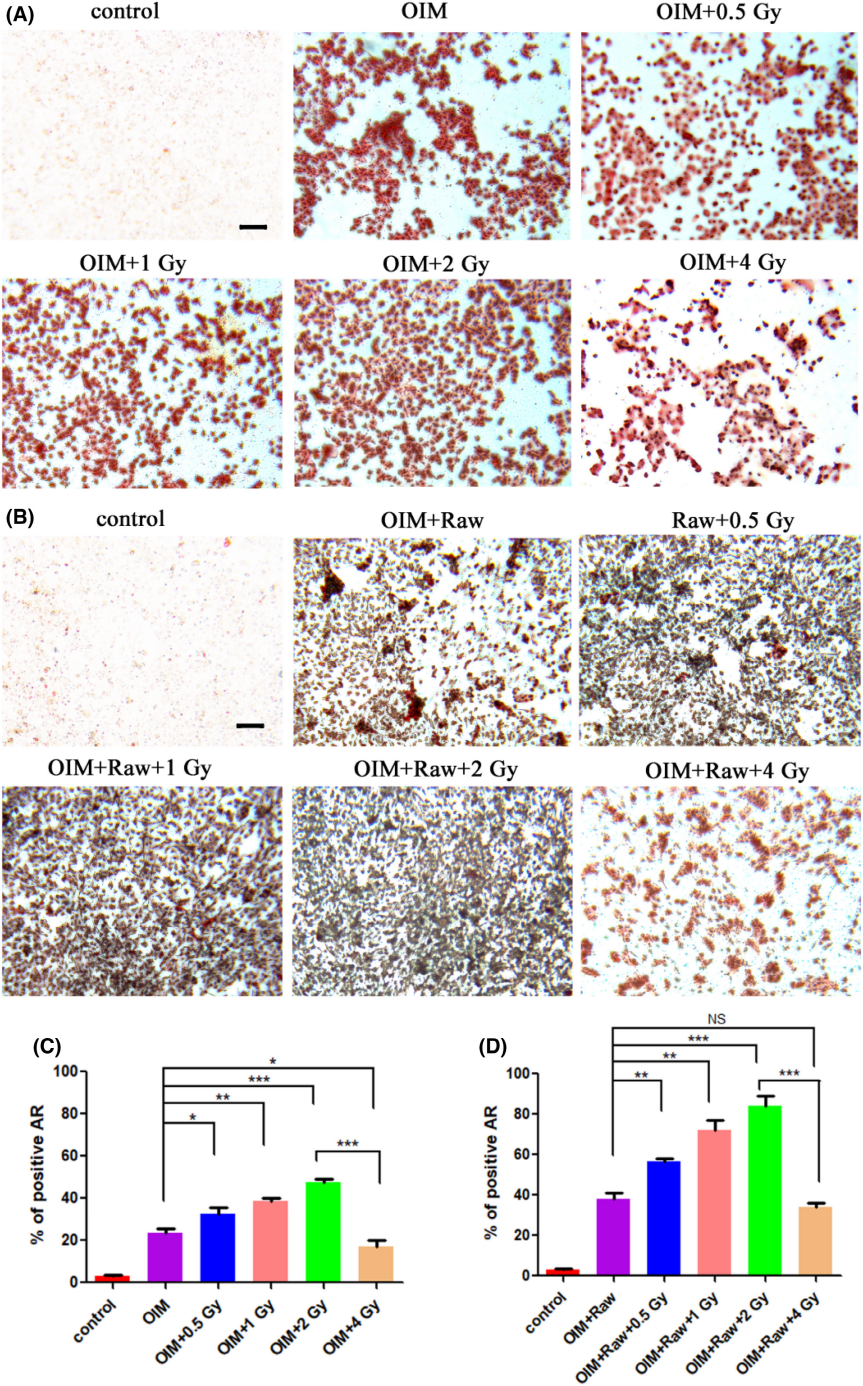
AR staining of BMSCs with different treatments at Day 21. (A) BMSCs treated with OIM and different doses of IR. (B) BMSCs treated with OIM, different doses of IR, and supernatants of Raw264.7 cells irradiated with corresponding doses of IR. (C) Semi‐quantitative of figures in (A). (D) Semi‐quantitative of figures in (B). Scale bar, 100 μm. NS: no significant difference, *p < 0.05, **p < 0.01, ***p < 0.001. The error bars in the graphs represent the standard deviation (*n* = 3).

Based on the above results, we concluded that IR ≤2 Gy can promote the proliferation of osteoblasts and induce M2 polarization of macrophages, while IR >2 Gy can inhibit the proliferation of osteoblasts and induce M1 polarization of macrophages. Does IR‐induced M2 polarization of macrophages promotes the proliferation of osteoblasts? To further explore it, the osteogenesis differentiation of BMSCs under different treatments were studied. BMSCs were cultured in OIM and then subjected to different doses of IR. After IR, one part of the BMSCs continued to be cultured, and the other part of cells were cultured with the supernatants of LPS‐pretreated Raw264.7 cells irradiated with corresponding doses of IR. Two osteogenesis markers, ALP (on Day 13) and mineralized nodules (on Day 21) were selected to reflect the osteogenic differentiation of BMSCs. As shown in Figures 3A,C and 4A,C, low‐dose IR ≤2 Gy exerted beneficial effects on bone repair as supported by the increased expression of ALP and mineralized nodules in BMSCs. The 2 Gy group showed the best performance with the highest level of ALP (49.8%) and AR (47.4%) staining compared with that in the OIM group (24.3%, 23.8%, respectively). While the 4 Gy group showed decreased expression of ALP (21.3%) and mineralized nodules (16.9%) compared with that in the OIM group, indicating that 4 Gy IR inhibited osteogenesis by inhibiting osteoblasts proliferation.

Moreover, after being supplied with the supernatants of Raw264.7 cells irradiated with corresponding doses of IR, the expression of ALP (Figure 3B,D) and mineralized nodules (Figure 4B,D) in BMSCs showed apparent increment. In line with the above observations, the addition of the supernatants of Raw264.7 cells irradiated with low‐dose IR ≤2 Gy significantly promoted the osteogenesis of BMSCs as the IR dose improved. The 2 Gy group showed the best performance with the highest level of ALP (88.7%) and AR staining (83.9%) compared with that in the OIM+Raw group (34.0%, 38.0%, respectively). As expected, incubation with the supernatant of Raw264.7 cells irradiated with 4 Gy of IR showed decreased expression of both ALP (27.5%) and mineralized nodules (34.3%) in BMSCs, suggesting the negative effect of IR >2 Gy in bone regeneration.

### 3.4 Expression of osteogenic genes

**FIGURE 5:**
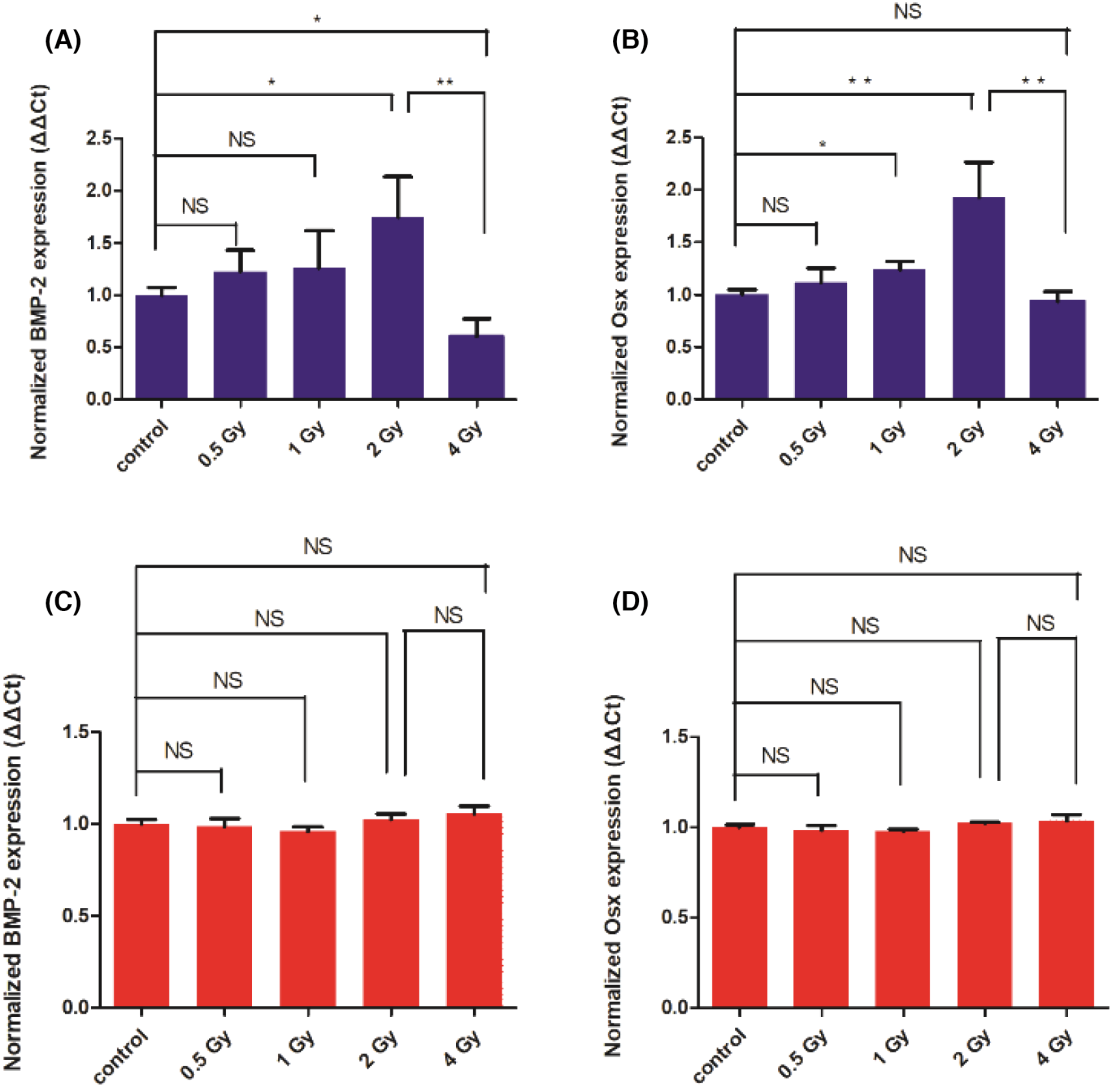
Expression of BMP‐2 and Osx in BMSCs after different treatments. The expression of BMP‐2 (A) and Osx (B) in BMSCs co‐cultured with the supernatants of Raw264.7 cells irradiated with different doses of IR. The expression of BMP‐2 (C) and Osx (D) in BMSCs co‐cultured with the supernatants of Raw264.7 cells, in which the Raw264.7 cells were irradiated with different doses of IR and then subjected to LPS stimulation. NS: no significant difference, *p < 0.05, **p < 0.01. The error bars in the graphs represent the standard deviation (*n* = 3).

To further verify the effect of macrophage polarization induced by different doses of IR on osteoblasts, qRT‐PCR was conducted to quantitatively detect the expression of osteogenic genes (BMP‐2, Osx) in BMSCs. First, LPS prestimulated Raw264.7 cells were irradiated with different doses of IR, and the supernatants were collected (supernatants 1).Then BMSCs were cocultured with the supernatants 1 for 7 days and conducted to qRT‐PCR detection. The results (Figure 5A,B) showed that compared with the group of BMSCs cocultured with unirradiated Raw264.7 supernatants (the control group), in the low‐dose IR group, the osteogenic gene expression of BMSCs cocultured with the supernatants of irradiated Raw264.7 increased remarkably with the increment of IR dose. The expression of BMP‐2 and Osx in the group of BMSCs cocultured with the supernatants of Raw264.7 irradiated with 1 Gy considerably up‐regulated to 1.26‐fold and 1.24‐fold, respectively. The 2 Gy group had the most remarkably increment, with the expression of BMP‐2 increased to 1.75‐fold, and the expression of Osx increased to 1.9‐fold. However, when the IR dose continued to increase to 4 Gy, the expression of BMP‐2 was reduced to 0.6‐fold of that in the control group. This indicates that M2 polarization of macrophages induced by low‐dose IR ≤2 Gy can indeed increase the expression of osteogenic genes in osteoblasts, while M1 polarization of macrophages induced by IR dose >2 Gy can reduce the expression of osteogenic genes. Then, how will the expression of osteogenic genes in osteoblasts change if M2 macrophages become M1 macrophages after inflammatory stimulation? To explore it, after suction of supernatant 1, the remaining cells in the plates were added with LPS (1 μg/mL) for 24 h, replaced with fresh culture medium for additional culture of 12 h, and supernatants 2 were also collected. The purpose of adding LPS again was re‐inflammatory stimulation of the irradiated macrophages to M1 macrophages. BMSCs were cocultured with the collected supernatants 2 in different groups. It was found (Figure 5C,D) that there is inconspicuous difference in the expression of BMP‐2 and Osx, which may be due to the M1 polarization of macrophages in all groups. This result indicates that the expression of osteogenic genes in BMSCs is closely related to the polarity of macrophages. In order to further demonstrate the osteogenic factor, nitric oxide, Pro‐inflammatory cytokine IL‐1β and anti‐inflammatory cytokine IL‐10 contents in supernatants 1 were detected. The results (Figure S3) showed that when the IR dose was gradually increased to 2 Gy, the contents of nitric oxide and IL‐1 were gradually decreased but the content of IL‐10 was gradually increased. However, when the IR dose increased to 4Gy, the contents of nitric oxide and IL‐1 increased significantly, and the content of IL‐10 decreased significantly. This proves that the changes in the relevant cytokines secreted by macrophages induced by IR mediate the osteogenic changes.

### 3.5 Low‐dose IR promotes bone regeneration in rat cranial defect model

**FIGURE 6:**
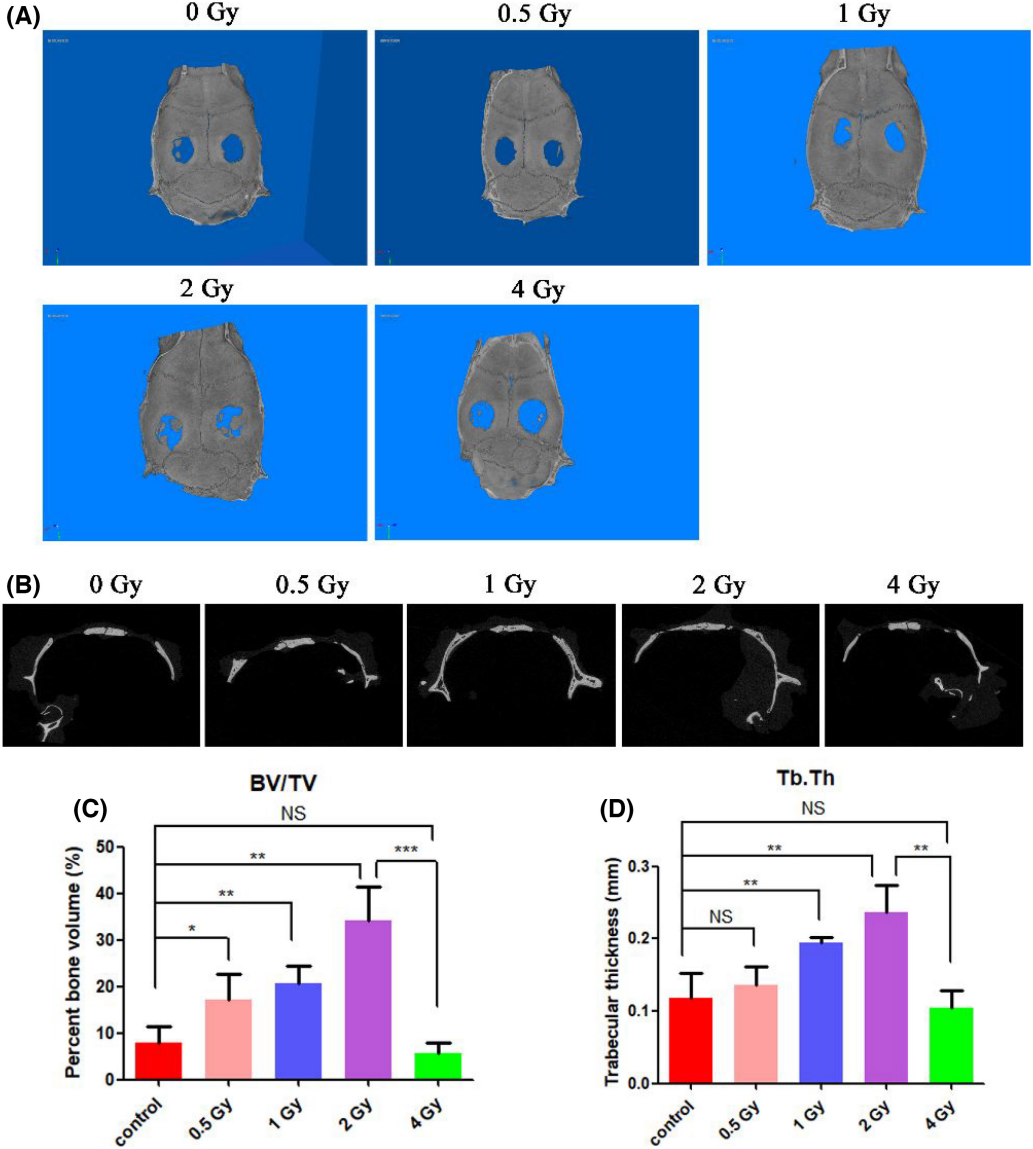
Micro‐CT analysis of the cranium after different doses of IR for 28 days. (A) 3D‐reconstruction of cranium in different groups. (B) Sagittal view of rat cranial defect in different groups. (C, D) Micro‐CT analysis of BV/TV (C) and Tb.Th (D). NS: no significant difference, *p < 0.05, **p < 0.01, ***p < 0.001. The error bars in the graphs represent the standard deviation (*n* = 3).

To reveal the effect of various doses of IR on bone regeneration, in our study, male SD rats with established cranial defects model were employed and subjected to 0.5–4 Gy of IR once. At 28 days post IR, micro‐CT was employed to analyse the formation of new bones within the defect region. As shown in Figure 6, the 3D‐reconstruction of the cranium in different groups (Figure 6A) revealed that when IR dose was ≤2 Gy, the amount of new bone increased with the increment of IR dose. Compared with the control group without IR, the amount of new bone in the 1 Gy group had a slight increase, and the amount of new bone in the 2 Gy group had the largest increase. In comparison, when the IR dose continued to increase to 4 Gy, it had no significant effect on bone regeneration, and no bone regeneration was observed along the edge of the defect. These observations were also verified by the sagittal view of rat cranial defect in Figure 6B. Quantification results (Figure 6C,D) demonstrated that compared with the control group without IR, the bone volume and trabecular thickness of the low‐dose IR ≤2 Gy groups were augmented when the IR dose was increased, and the bone volume and trabecular thickness of the 2 Gy group could reach 34.1 ± 7.3%, 0.236 ± 0.038 mm, respectively. However, when the IR dose increased to 4 Gy, the bone volume was only 5.5 ± 2.3% and trabecular thickness was only 0.104 ± 0.023 mm. The micro‐CT results indicate that low‐dose IR ≤2 Gy promotes bone regeneration in a dose‐dependent manner, the effect of bone regeneration was enhanced with the increase of IR dose, while IR >2 Gy has inconspicuous effect on bone regeneration.

**FIGURE 7:**
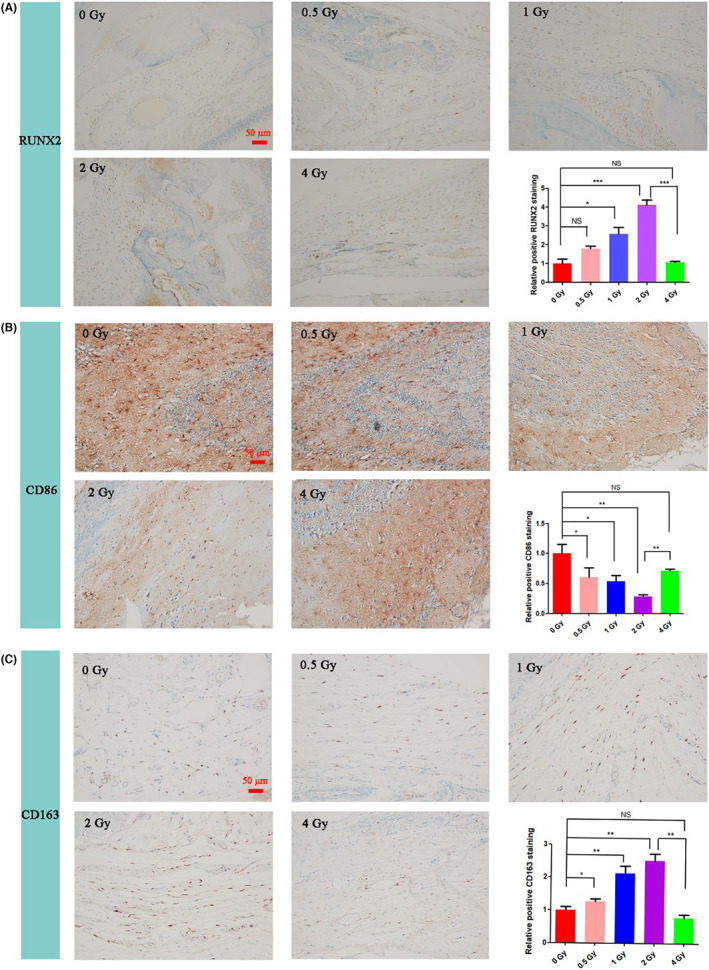
Immunohistochemical staining and semiquantitative analysis by imageJ software of RUNX2 (A), CD86 (B) and CD163 (C) in cranium after different doses of IR for 28 days. Brown precipitation represents positive staining. Scale bars, 50 μm. NS: no significant difference, *p < 0.05, **p < 0.01, ***p < 0.001. The error bars in the graphs represent the standard deviation (*n* = 3).

To investigate the expression of osteogenic markers and polarization markers following IR, immunohistochemical staining of osteogenic marker RUNX2, M1 polarization marker CD86 and M2 polarization marker CD163 were performed on the periphery of cranial defect samples in different groups on the 28th day after various doses of IR. As shown in Figure 7A, when the IR dose was ≤2 Gy, positive staining of RUNX2 was increased as the increment of the IR dose. Compared with the 0 Gy control group, the RUNX2 positive rate in the 0.5 Gy group was 1.49‐fold that of the control group, but there was no significant difference between the two groups. The RUNX2 positive rate in the 0.5 Gy group was 2.54‐fold that of the control group. The positive rate of RUNX2 in the 2 Gy group was significantly elevated, which was 3.60‐fold that of the control group. In contrast, further increase of IR dose to 4 Gy resulted in insignificant changes in RUNX2 expression compared with the control group. Immunohistochemical staining results reveal the osteoinductive ability of low‐dose IR ≤2 Gy by upregulating the expression of osteogenic markers, while by contrast, high‐dose IR >2 Gy has no noticeable bone regeneration effect with inconspicuous regulating of the osteogenic marker expression. As revealed in Figure 7B,C, when the IR dose was ≤2 Gy, the expression of CD86 decreased and the expression of CD163 increased with the increase of IR dose. Compared with the 0 Gy control group, the relative positive expression of CD86 in the 0.5 Gy group decreased to 0.6‐fold and the expression of CD163 increased to 1.27‐fold. The expression of CD86 in the 1 Gy group decreased to 0.54‐fold, and the expression of CD163 increased to 2.13‐fold. The 2 Gy group had the most significant change, with the expression of CD86 decreased to 0.28‐fold, and the expression of CD163 increased to 2.51‐fold. When the IR dose continued to increase to 4 Gy, the expression of CD86 and CD163 had no significant difference compared with the control group. This result indicates that low‐dose IR ≤2 Gy can promote the M2 polarization of macrophages in the cranial defect site and reduce the M2 polarization, while IR >2 Gy has the opposite effect.

### 3.6 Low‐dose IR promotes M2 polarization of peripheral blood monocytes in vivo

**FIGURE 8:**
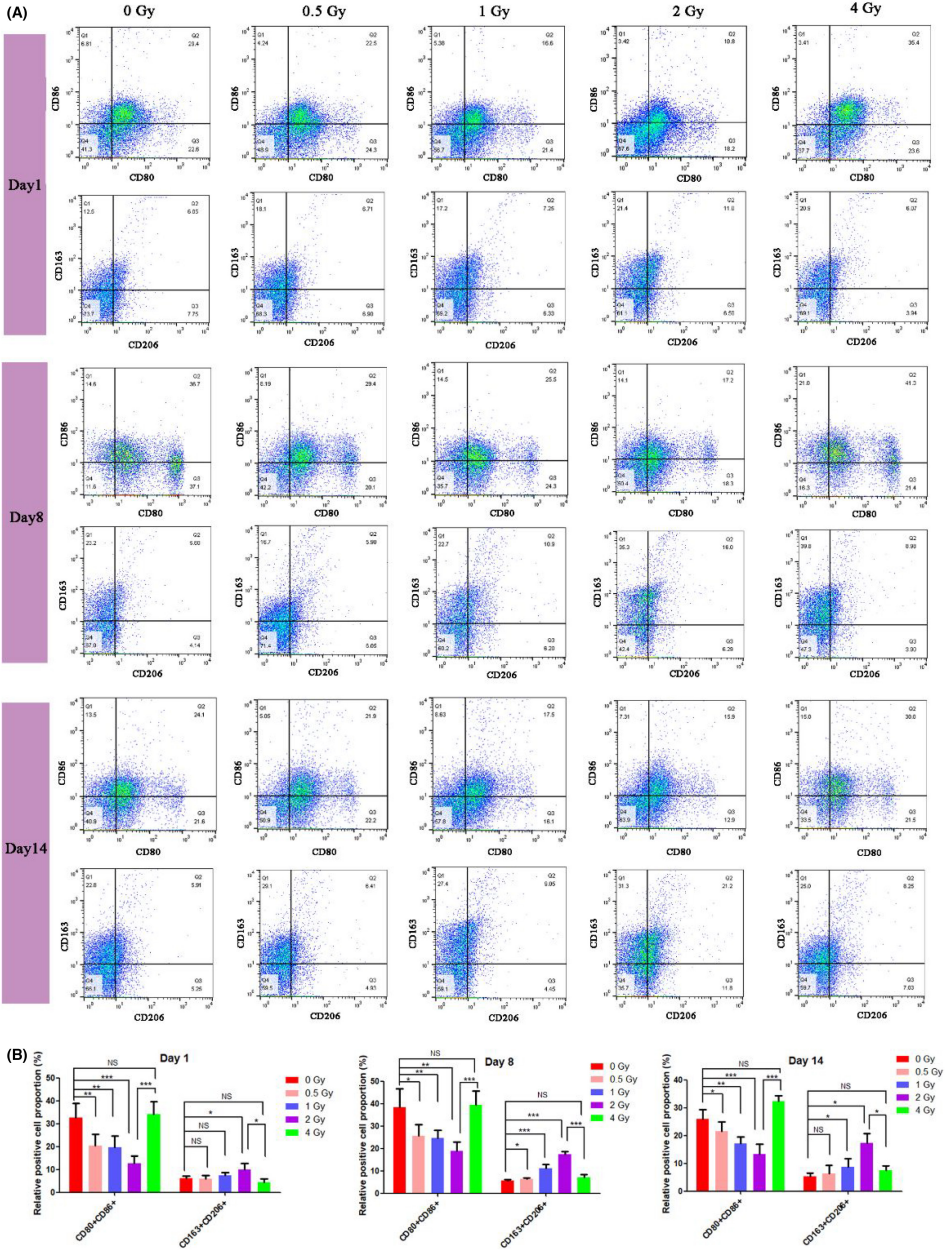
Flow cytometric results (A) of peripheral blood monocytes M1 (CD80, CD86) and M2 (CD163, CD206) markers from the blood samples of the cranial defect rats and corresponding quantitative results (B) on Days 1, 8 and 14 after IR. NS: no significant difference, *p < 0.05, **p < 0.01, ***p < 0.001. The error bars in the graphs represent the standard deviation (*n* = 5).

In order to further verify the effect of IR on the polarization of peripheral blood monocytes in cranial defect rats, the orbital blood of rats after various doses of IR (0–4 Gy) at a predetermined time (Day 1, 8, 14) were collected. Then PBMC was isolated and four M1/M2 antibodies (CD80/CD86 for M1‐polarized monocytes and CD206/CD163 for M2‐polarized monocytes) were used as the marks of monocytes polarity, and the samples were detected by flow cytometer. It showed on Day 1 (Figure 8A), for the low‐dose IR ≤2 Gy groups, with the increase of IR dose, the proportion of CD80^+^CD86^+^ monocytes decreased, and that of CD163^+^CD206^+^ monocytes increased. In the 0 Gy control group (Figure 8B, Day 1), the proportion of CD80^+^CD86^+^ and CD163^+^CD206^+^ monocytes were 32.7% and 6.2%, respectively, while in the 2 Gy group, the proportion of CD80^+^CD86^+^ monocytes reduced to 12.8%, and the proportion of CD163^+^CD206^+^ monocytes increased to 10.3%. When the IR dose comes to 4 Gy, the proportion of CD80^+^CD86^+^ monocytes instead increased to 34.07%, and the proportion of CD163^+^CD206^+^ monocytes instead decreased to 4.72%. On Day 8, in the 0 Gy group (Figure 8B, Day 8), the proportion of CD80^+^CD86^+^ and CD163^+^CD206^+^ monocytes were 38.3% and 5.6%, respectively, while in the 2 Gy group, the proportion of CD80^+^CD86^+^ monocytes reduced to 18.8%, and the proportion of CD163^+^CD206^+^ monocytes increased to 17.3%. For the 4 Gy group, the proportion of CD80^+^CD86^+^ monocytes instead increased to 39.4%, and the proportion of CD163^+^CD206^+^ monocytes instead decreased to 6.6%. Day 14 showed a similar trend. On Day 14, for the low‐dose IR ≤2 Gy groups, when the IR of 0 Gy gradually increased to 0.5, 1, 2 Gy, the proportion of CD80^+^CD86^+^ monocytes gradually decreased from 25.9% to 13.4%, while the proportion of CD163^+^CD206^+^ monocytes gradually increased from 1.3% to 17.4%. When the IR dose continued to increase to 4 Gy, the proportion of CD80^+^CD86^+^ monocytes increased to 32.3%, and the proportion of CD163^+^CD206^+^ monocytes decreased to 7.5%. These results indicate that low‐dose IR ≤2 Gy can increase the ratio of M2‐polarized monocytes and conversely decrease that of M1‐polarized ones in peripheral blood. In contrast, from 2 to 4 Gy, IR reduced the ratio of M2‐polarized monocytes but increased that of M1‐polarized ones in peripheral blood, which exhibited the opposite result.

The effect of IR on the polarization of monocytes in healthy rats was also studied (Figure S4). Healthy rats showed a similar polarization trend as that in cranial defect rats on Days 1 and 3. Interestingly, the irradiated healthy rats showed faster recovery of the changed polarization state (Figure S4) as compared with the cranial defect model. It was shown that the polarization differences among different groups were started to unify in healthy rats at Day 14, and there were almost no differences at Day 28. This may be due to the fact that healthy body/organ has a better ability to adjust body indicators to a normal state, which deserves our future exploration.

## 4 CONCLUSIONS

In this study, we found that low‐dose IR ≤2 Gy showed enhanced proliferation of BMSCs, while IR >2 Gy showed impaired cell viability. Low‐dose IR ≤2 Gy induced M2 polarization of macrophages, while IR >2 Gy was more prone to enhance M1 polarization of macrophages both in vitro and in vivo experiments. Low‐dose IR ≤2 Gy promotes bone regeneration not only by directly promoting the proliferation of osteoblasts, but also by triggering M2 polarization of macrophages to promote bone regeneration. The immunologic mechanism and application of low‐dose IR on bone regeneration still needs further study and discussion.

## AUTHOR CONTRIBUTIONS

**Shaoqing Chen:** Conceptualization (equal); data curation (equal); investigation (equal); software (equal); writing – original draft (equal). **Su Ni:** Conceptualization (equal); data curation (equal); investigation (equal); software (equal); supervision (equal). **Chun Liu:** Data curation (equal); methodology (equal); software (equal). **Mu He:** Data curation (equal); investigation (equal); software (equal). **Yiwen Pan:** Data curation (equal); methodology (equal); software (equal). **Pengfei Cui:** Data curation (supporting); software (equal). **Cheng Wang:** Data curation (supporting); software (supporting). **Xinye Ni:** Conceptualization (lead); funding acquisition (lead); resources (lead); writing – review and editing (lead).

## FUNDING INFORMATION

This work has been supported by National Natural Science Foundation of China under Grant No. 81871756; Innovation Fund of National Orthopaedics and Exercise Rehabilitation Clinical Medical Research Center, research number: 2021‐NCRC‐CXJJ‐ZH‐13; Jiangsu Provincial Medical Key Discipline Cultivation Unit (JSDW202237); Jiangsu Provincial Key Research and Development Program Social Development Project (BE2022720); General Program of Jiangsu Provincial Health Commission (M2020006); Jiangsu Provincial Outstanding Postdoctoral Program (2022ZB824); Leading Innovative Talent Introduction and Cultivation Project of Changzhou “Dragon City Talent Plan” (CQ20220107); Science and Technology Project for Youth Talent of Changzhou Health Commission (QN202224).

## CONFLICT OF INTEREST STATEMENT

The authors declare no conflict of interest.

## Supporting information


Appendix S1

## Data Availability

All materials and data supporting this study are available from the author (chenshaoqing@zju.edu.cn) upon reasonable request.
