# Supplementary material for: Neglected immunoregulation: M2 polarization of macrophages triggered by low‐dose irradiation plays an important role in bone regeneration
Source: J Cell Mol Med. 2023 Mar 16;27(8):1095–109. doi: 10.1111/jcmm.17721 (PMC10098298; doi:10.1111/jcmm.17721)
Supplement: Supplementary file 1 — Appendix S1 [file JCMM-27-1095-s001.docx]

Neglected immunoregulation: M2 polarization of macrophages triggered by low-dose irradiation plays an important role in bone regeneration

Shaoqing Chen^‡1,2^, Su Ni^‡1^, Chun Liu^1^, Mu He^1^, Yiwen Pan^1,3^, Pengfei Cui^3^, Cheng Wang^3^, Xinye Ni^*1,2^

1 The Affiliated Changzhou No. 2 People's Hospital of Nanjing Medical University, Changzhou, Jiangsu 213003, China.

2 Jiangsu Province Engineering Research Center of Medical Physics, Jiangsu 213003, China.

3 School of Pharmacy, Changzhou University, Changzhou, Jiangsu 213164, China.

‡ These authors contributed equally to this work.

*****Corresponding author: Xinye Ni, nxy@njmu.edu.cn

Table S1 Primers used for RT-PCR

Gene Primer Sequence

IL-1β Forward TCGCAGCAGCACATCAACAAGAG

Reverse AGGTCCACGGGAAAGACACAGG

iNOS Forward CTGCAGCACTTGGATCAGGAACCTG

Reverse GGAGTAGCCTGTGTGCACCTGGAA

BMP2 Forward GGGACCCGCTGTCTTCTAGT

Reverse TCAACTCAAATTCGCTGAGGAC

CD206 Forward AGGACGAAAGGCGGGATG

Reverse TTGGGTTCAGGAGTTGTTGTG

GADPH Forward AGGTCGGTGTGAACGGATTTG

Reverse TGTAGACCATGTAGTTGAGGTCA


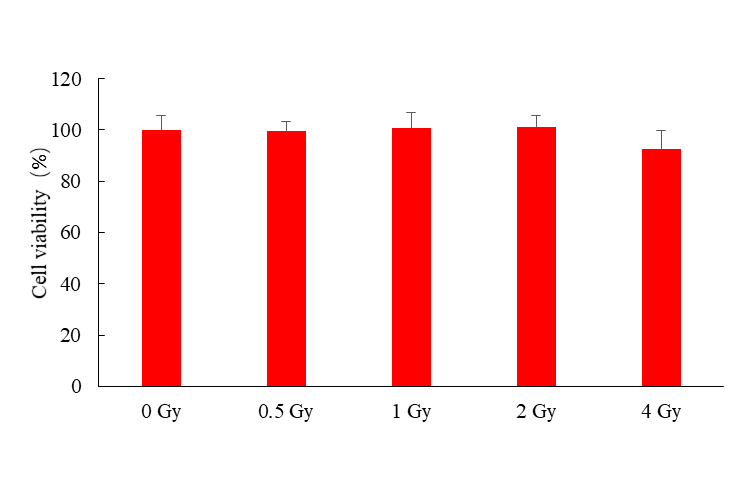


Figure S1 MTT assay of Raw264.7 cells treated with different doses of IR.


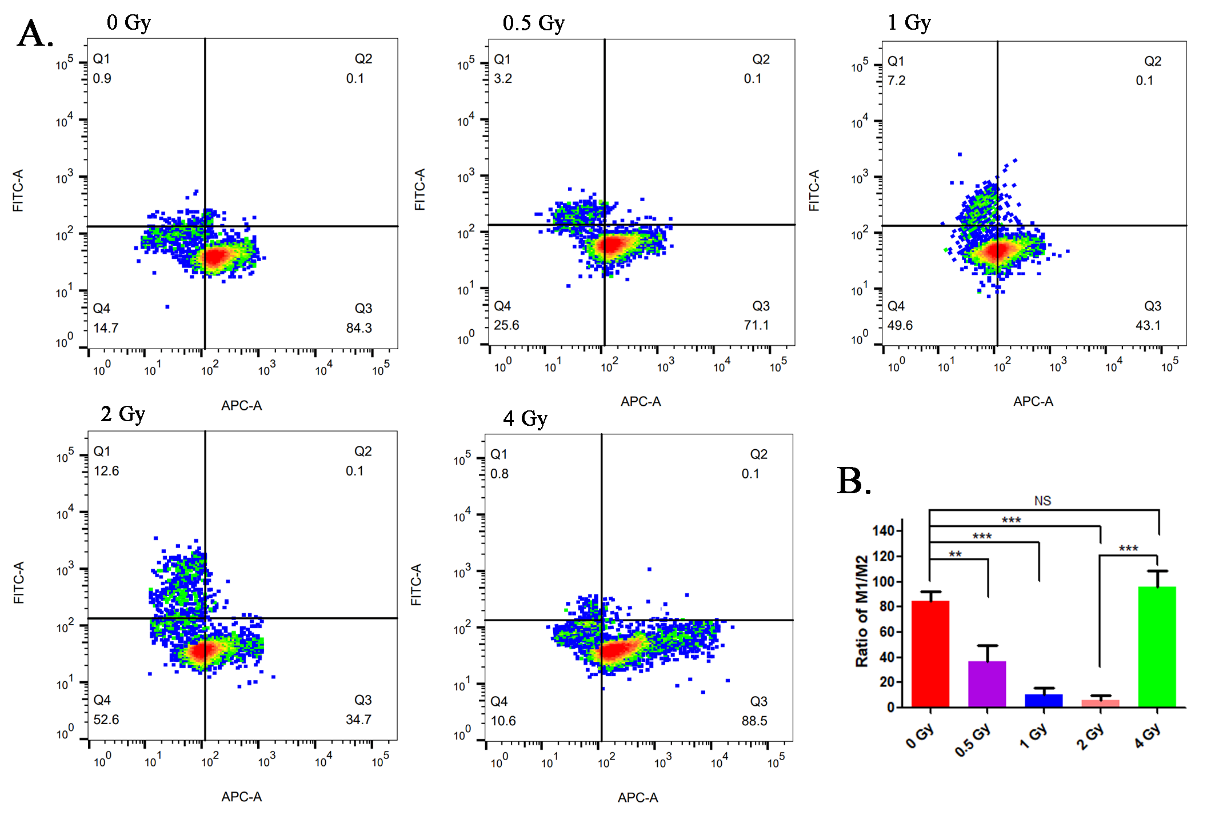


Figure S2 Flow cytometry assay of the proportion of M1/M2 in Raw264.7 cells at the time the supernatants were collected (A) and ratio of M1/M2 macrophages. APC: CD86, FITC: CD206.


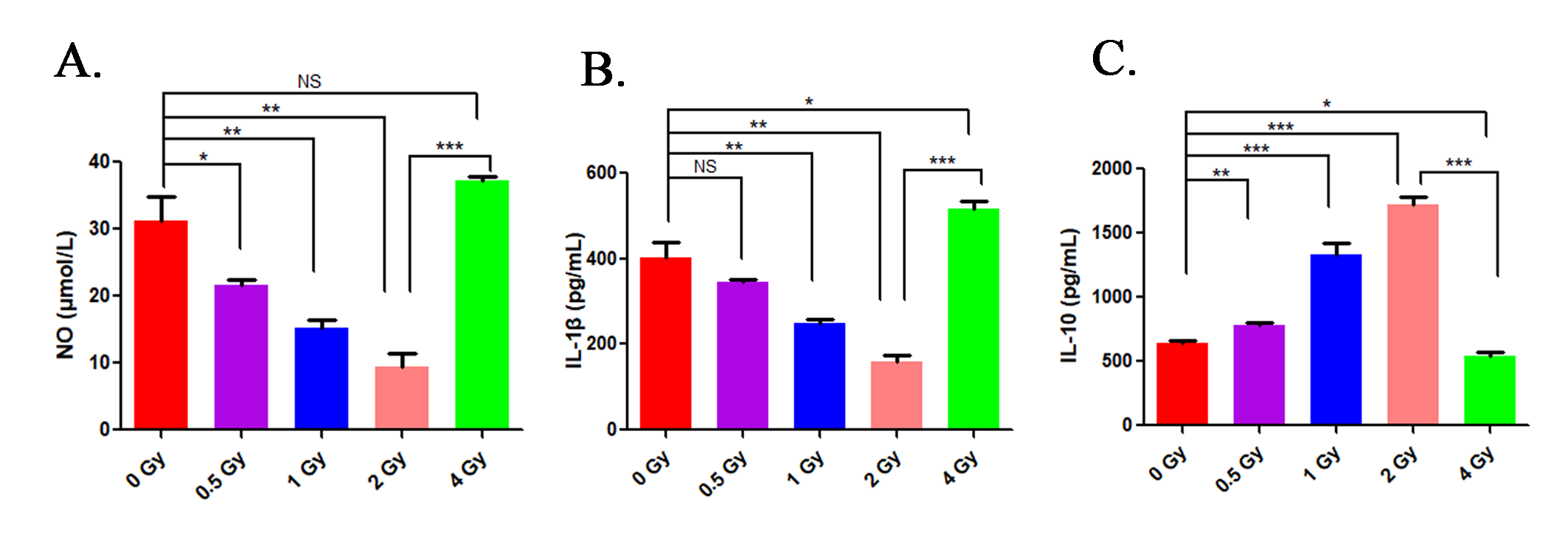


Figure S3 Contents of NO, IL-1 and IL-10 in the culture supernatant of LPS-pretreated Raw264.7 cells irradiated with different doses of IR. NS: no significant difference, *P<0.05, **P<0.01, ***P<0.001.


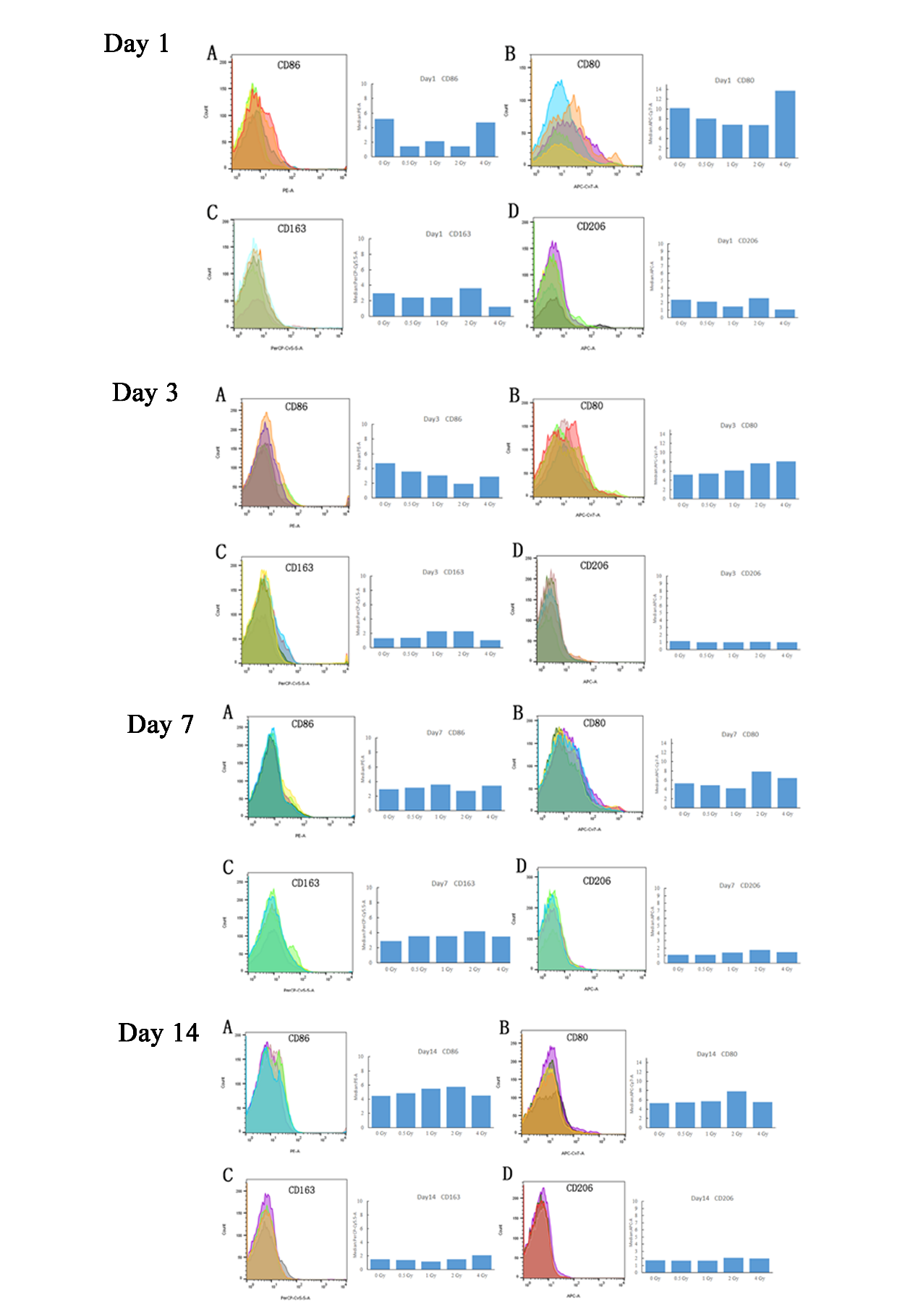


Figure S4 Flow cytometric results of macrophage M1 and M2 markers from the blood samples of the healthy rats on Day1 (A), Day3 (B), Day7 (C) and Day14 (D) after IR.
